# Multiple routes for non-physiological l-threonine uptake in *Escherichia coli* K-12

**DOI:** 10.3389/fmicb.2025.1579813

**Published:** 2025-04-03

**Authors:** Dmitrii M. Bubnov, Andrey A. Khozov, Tatiana V. Vybornaya, Agnessa A. Stepanova, Sergey V. Molev, Olga E. Melkina, Gennadii A. Badun, Maria G. Chernysheva, Ilia A. Skob, Alexander I. Netrusov, Sergey P. Sineoky

**Affiliations:** ^1^National Research Centre “Kurchatov Institute”, Moscow, Russia; ^2^Department of Microbiology, Faculty of Biology, Lomonosov Moscow State University, Moscow, Russia; ^3^Department of Radiochemistry, Faculty of Chemistry, Lomonosov Moscow State University, Moscow, Russia; ^4^Department of Genetics, Faculty of Biology, Lomonosov Moscow State University, Moscow, Russia

**Keywords:** *Escherichia coli*, l-threonine uptake, transmembrane transport, amino acid transporter, membrane proteins

## Abstract

In this study, we identified eight multicopy suppressors (*yhjE, sdaC, ydgI, alaE, ychE, yqeG, proP*, and *yjeM*) and three distinct classes of chromosomal mutations (*lrp, marC*, and *cycA*) capable of complementing the growth defect caused by threonine uptake deficiency in the *sstT tdcC livKHMGF brnQ thrP* strain. YhjE, SdaC, YdgI, AlaE, mutant MarC, and CycA exhibited measurable threonine-specific uptake activity in the *in vitro* assay. Phenotypic assays revealed that YhjE and SdaC were the main entry points for threonine in a strain lacking major threonine-specific permeases. A derivative of the threonine-auxotrophic *sstT tdcC livKHMGF brnQ thrP* mutant, harboring deletions of eight multicopy suppressors, exhibited significantly reduced fitness at subsaturating threonine concentrations and improved fitness at toxic threonine concentrations, indicating a defect in membrane permeability. These results may help guide the effective construction of threonine-producing strains, extend knowledge on the substrate preferences of SdaC, AlaE, and ProP, and provide clues for further studies on the exact substrate range of YhjE, YdgI, YjeM, YchE, MarC, and YqeG whose physiologically relevant functions have not yet been established.

## 1 Introduction

The transport of metabolites across the cytoplasmic membrane is a process that plays a pivotal role in the assimilation of carbon and energy sources, as well as other compounds required for biosynthetic reactions. This cellular subsystem mediates the interactions between cells within a population and provides an adequate response to variations in the composition of the surrounding environment. The mechanisms of solute uptake are of great interest in clinical practice, with recent studies showing that functional amino acid transport systems are essential virulence factors for the severe human pathogens *Yersinia pestis* and *Bacillus anthracis* (Palace et al., 2014; Dutta et al., 2022). Hence, uptake systems are promising targets for the production of live-attenuated vaccines and new antibacterial drugs. Membrane transport systems are primary targets for the development of industrial strains capable of producing valuable chemicals, especially amino acids. Inactivation of membrane proteins that facilitate the uptake of a desired compound and overexpression of appropriate exporters promote their excretion into the medium, thereby preventing feedback inhibition of key biosynthetic enzymes and toxicity to producer cells (Okamoto et al., 1997; Kruse et al., 2001, 2002; Doroshenko et al., 2007; Park et al., 2007; Mundhada et al., 2016). Therefore, the mechanisms of transmembrane transport have been extensively studied over the past few decades. However, the relevant functions of the various membrane proteins remain unclear. For instance, the “*y*-om” of *Escherichia coli*, which is a set of genes that lack experimental evidence of function, contains 295 genes encoding putative membrane transporters (Ghatak et al., 2019), whose substrate specificity and physiologically relevant functions remain unknown.

In our previous study, we identified and characterized a high-affinity permease, ThrP, which is capable of translocating l-threonine and l-serine across the cytoplasmic membrane of *E. coli* K-12 (Khozov et al., 2023). The results suggest that the LIV-I system also participates in l-threonine transport while having *K*_M_ and *V*_max_ similar to those of ThrP. Additionally, we found that BrnQ, a dedicated transporter for l-isoleucine, l-valine, and l-leucine, is active toward l-threonine under normal conditions and is a primary l-threonine permease at unphysiologically high substrate concentrations. Together with the previously described l-threonine transporters SstT and TdcC (Hama et al., 1987; Sumantran et al., 1990; Ogawa et al., 1997), these proteins confer a major fraction of the l-threonine uptake activity detectable at substrate concentrations of 50–100 μM. Meanwhile, data on the phenotype of a mutant strain auxotrophic for l-threonine and lacking all the listed transport systems suggested that the cell could still consume l-threonine from the medium when its concentration was increased to 1.0–2.0 mM. This observation led us to presume that the cell possessed unknown transport systems that exhibited low-affinity l-threonine transport activity.

In the present study, we identified several permeases via screening of multicopy suppressors and genomic suppressor mutations that complement the growth defect of a strain lacking the major l-threonine transport systems. The results shed light on the physiologically relevant function of several “*y*”-genes and provide an approach for further elucidation of their role in *E. coli* cell biology. This study also extends the substrate range of well-established amino acid carriers.

## 2 Materials and methods

### 2.1 Bacterial strains and plasmids

The bacterial strains used in this study were derived from *E. coli* K-12 MG1655. The genotypes and relevant characteristics of all strains and plasmids used are listed in Tables 1, 2, respectively. The strains were constructed using a combination of λRed-mediated recombineering (Datsenko and Wanner, 2000) and P1 transduction (Thomason et al., 2007). The pBR-*yhjE*, pBR*-yjeM*, pBR*-sdaC*, pBR*-ydgI*, pBR*-alaE*, pBR*-ychE*, pBR-*yqeG*, and pBR*-proP* plasmids were recovered from a genomic library of the B1426 strain, based on their ability to enable the growth of the *sstT tdcC thrP livKHMGF brnQ* mutant B1895 strain on M9 agar supplemented with 0.2 g/L glucose and 1 mM threonine. The exact coordinates (according to the genome of the MG1655 strain; GenBank accession number U00096.3) of the genomic inserts found within the plasmids upon sequencing are listed in Table 2. The pDEW_*yhjE* reporter plasmid was constructed by ligating two DNA fragments. The insert was amplified from the chromosome of MG1655 using primers yhjE_p_F (5′-ATCGATGAATTCAATAGGCCGGATGCGGCG-3′) and yhjE_p_R (5- GAGCTCGGTACCCGGTTATTTTTTGGCTAACGAATAGC-3′). The resulting fragment was digested using KpnI and EcoRI and ligated into the pDEW201 vector (Van Dyk and Rosson, 1998) that was linearized using the same endonucleases.

**Table 1:** Bacterial strains used in this study.

Strain	Genotype	Source
MG1655	F^−^ λ^−^ *ilvG^−^ rfb-50 rph-1*	Laboratory collection
B1426	MG1655 *∆thrBC ∆sstT ∆tdcBCDE::neo*	
B1895	MG1655 *∆thrBC ∆sstT ∆tdcBCDE::neo ΔthrP ΔbrnQ ∆livKHMGF::cat*	This study
B1950	MG1655 *∆thrBC ∆sstT ∆tdcBCDE::neo ΔthrP ΔbrnQ ∆livKHMGF ∆yjeM ∆sdaC ∆ydgI ∆ychE*	
B2055	B1950 *lrp*^T134A^	
B2058	B1950 *marC*^S140SS^	
B2059	B1950 *marC*^V145E^	
B2061	B1950 *cycA*^V226A^	
B2063	B1950 *marC*^L10Q^	
B2068	B1950 *marC*^G11E^	
B2071	B1950 *cycA*^C110S^	
B2394	MG1655 *∆sstT ∆tdcBCDE::neo ΔthrP ΔbrnQ ∆livKHMGF*	
B2722	MG1655 *∆thrBC ∆sstT ∆tdcBCDE::neo ΔthrP ΔbrnQ ∆livKHMGF ∆yhjE::cat*	
B2769	MG1655 *∆thrBC ∆sstT ∆tdcBCDE::neo ΔthrP ΔbrnQ ∆livKHMGF ∆yhjE::cat ∆yjeM::aadA1*	
B2789	MG1655 *∆thrBC ∆sstT ∆tdcBCDE::neo ΔthrP ΔbrnQ ∆livKHMGF ∆yhjE ∆yjeM ∆alaE::aadA1*	
B2792	MG1655 *∆thrBC ∆sstT ∆tdcBCDE::neo ΔthrP ΔbrnQ ∆livKHMGF ∆yhjE ∆yjeM ∆alaE::aadA1 ∆proP::cat*	
B2794	MG1655 *∆thrBC ∆sstT ∆tdcBCDE::neo ΔthrP ΔbrnQ ∆livKHMGF ∆yhjE ∆yjeM ∆alaE ∆proP ∆sdaC::aadA1*	
B2797	MG1655 *∆thrBC ∆sstT ∆tdcBCDE::neo ΔthrP ΔbrnQ ∆livKHMGF ∆yhjE ∆yjeM ∆alaE ∆proP ∆sdaC ∆ychE::aadA1*	
B2800	MG1655 *∆thrBC ∆sstT ∆tdcBCDE::neo ΔthrP ΔbrnQ ∆livKHMGF ∆yhjE ∆yjeM ∆alaE ∆proP ∆sdaC ∆ychE ∆ydgI::aadA1*	
B2818	MG1655 *∆thrBC ∆sstT ∆tdcBCDE::neo ΔthrP ΔbrnQ ∆livKHMGF ∆yhjE ∆yjeM ∆alaE ∆proP ∆sdaC ∆ychE ∆ydgI ∆yqeG::aadA1*	
B2820	B2055 *∆lrp*	
B2824	B2055 ∆*yhjE*::*aadA1*	
B2827	B2055 *∆yhjE ∆proP::cat*	
B2873	B2055 ∆*yhjE ∆alaE::aadA1*	
B2875	B2055 ∆*yhjE ∆proP::cat ∆yqeG::aadA1*	

**Table 2:** Plasmids used in this study.

Plasmids	Relevant characteristics (coordinates are in accordance with the U00096.3 genbank entry)	Source
pBR322	Contains the pMB1 origin of replication and the *bla* (Ap^R^), and *tetA* (Tc^R^) markers; medium-copy-number cloning vector.	Bolivar et al. (1977)
pDEW201	Contains the pMB1 origin of replication, the *bla* (Ap^R^) marker, and the promoterless *luxCDABE* operon of *Photorhabdus luminescens*; promoter probe vector.	Van Dyk and Rosson (1998)
pDEW_*yhjE*	A derivative of pDEW201, carrying the promoter of *yhjE* upstream of the *luxCDABE* operon.	This study
pBR-*ychE*	A derivative of pBR322, carrying the region of *E. coli* K-12 chromosome corresponding to coordinates 1,297,744–1,301,027 inserted into the BamHI site. The insert contains the *ychE* ORF.	
pBR-*yjeM*	A derivative of pBR322, carrying the region of *E. coli* K-12 chromosome corresponding to coordinates 4,383,331–4,385,560 inserted into the BamHI site. The insert contains the *yjeM* ORF.	
pBR-*yhjE*	A derivative of pBR322, carrying the region of *E. coli* K-12 chromosome corresponding to coordinates 3,673,707–3,676,353 inserted into the BamHI site. The insert contains the *yhjE* ORF.	
pBR-*alaE*	A derivative of pBR322, carrying the region of *E. coli* K-12 chromosome corresponding to coordinates 2,798,554–2,800,143 inserted into the BamHI site. The insert contains the *ygaC* ORF additionally to *alaE.*	
pBR-*proP*	A derivative of pBR322, carrying the region of *E. coli* K-12 chromosome corresponding to coordinates 4,328,041–4,332,207 inserted into the BamHI site. The insert contains the *rdcB* and *pmrR* ORFs additionally to *proP.*	
pBR-*yqeG*	A derivative of pBR322, carrying the region of *E. coli* K-12 chromosome corresponding to coordinates 2,984,996–2,987,940 inserted into the BamHI site. The insert contains the *yqeG* ORF.	
pBR-*ydgI*	A derivative of pBR322, carrying the region of *E. coli* K-12 chromosome corresponding to coordinates 1,679,357–1,681,299 inserted into the BamHI site. The insert contains the *ydgI* ORF.	
pBR-*sdaC*	A derivative of pBR322, carrying the region of *E. coli* K-12 chromosome corresponding to coordinates 2,927,439–2,930,367 inserted into the BamHI site. The insert contains the *sdaC* ORF.	

### 2.2 Media and culture conditions

Bacteria were routinely grown in lysogeny broth (LB) (10 g/L tryptone, 5 g/L yeast extract, and 10 g/L sodium chloride) at 37°C with shaking at 220 rpm. A solid LB medium was prepared by adding 20 g/L agar to the LB medium. Ampicillin (200 μg/mL), kanamycin (100 μg/mL), spectinomycin (50 μg/mL), and chloramphenicol (20 μg/mL) were added to the medium as needed. Either solid or liquid M9 minimal medium (Sambrook et al., 1989) with 0.2% glucose was used for the phenotype and transport assays. The medium was supplemented with l-threonine, as indicated in the Results section and figure legends. Phenotypic assays were performed as previously described (Khozov et al., 2023).

### 2.3 DNA manipulations

Standard methods were used for chromosomal DNA isolation, restriction enzyme digestion, agarose gel electrophoresis, ligation, and transformation (Sambrook et al., 1989). PCR amplification was performed using DreamTaq (Thermo Fisher Scientific, Vilnius, Lithuania) or KAPA HIFI (Kapa Biosystems, Wilmington, MA, United States) polymerase. Plasmids were isolated and DNA fragments were extracted using GeneJET Plasmid Miniprep and GeneJET Gel Extraction kits (Thermo Fisher Scientific).

### 2.4 Identification of multicopy suppressors of the growth defect caused by the *sstT tdcC thrP livKHMGF brnQ* mutations

A genomic library of the *sstT tdcC thrBC* mutant B1426 strain was prepared using the pBR322 vector, as described previously (Khozov et al., 2023). The library was electroporated into the B1895 strain, and transformants were selected on M9 agar plates supplemented with 200 mg/L ampicillin and 1 mM threonine and incubated for 3 days. The resulting colonies were repurified on the same medium to be used for plasmid isolation, followed by sequencing of the genomic inserts using the following primers: 5′- GGTTGAGGCCGTTGAGCAC-3′ and 5′- ACATTAACCTATAAAAATAGGCG-3′.

### 2.5 Identification of chromosomal mutations that suppress threonine uptake defect

First, several independent cultures of the B1950 strain were inoculated by picking single colonies into 50 mL test tubes with 20 mL of LB medium, which were incubated overnight with shaking at 37°C and 220 rpm. The next day, each culture was washed twice with 50 mL of 0.9% NaCl solution. The cells were concentrated 10 times relative to the original volume, after which 100 μL aliquots were plated onto M9 agar plates supplemented with 0.2 g/L glucose and 400 μM threonine and incubated at 37°C for 2 days. The resulting mutants were picked, restreaked on the same medium, and subjected to genome resequencing on an Illumina MiSeq (Illumina, CA, United States). Paired-end 2 × 250 bp reads were aligned against the reference genome of the MG1655 strain (U00096.3 GenBank entry) using Breseq 0.36.1 (Barrick et al., 2014) in consensus mode. A set of common mutations detected in the genomes of all the analyzed strains was considered to be inherited from the parental strain and excluded from further analysis.

### 2.6 Threonine uptake assay

Uniformly labeled l-[U-^14^C]threonine was obtained from Moravek Biochemicals (United States). To measure l-[U-^14^C]threonine uptake, cells were grown overnight in 5 mL of M9 medium supplemented with 0.2% glucose at 37°C with shaking at 220 rpm. Plasmid-carrying strains were grown in the presence of 100 mg/L ampicillin, whereas the other strains were incubated in antibiotic-free M9 medium. For threonine-auxotrophic strains, the medium was supplemented with 50 mM threonine. The overnight culture was diluted in 20 mL of the same medium to obtain an OD_600_ of 0.0625 and was grown until the OD_600_ reached 0.5. The cells were harvested in 50 mL polypropylene tubes via centrifugation at 5,000 × *g* at 4°C for 5 min. The supernatant was discarded, and the cells were washed once with 35 mL of M9 medium. The cells were resuspended in 10 mL of M9 medium with 0.2% glucose and incubated for 2 h at 37°C with shaking at 220 rpm to ensure intracellular threonine exhaustion. All subsequent steps were performed on ice. The cells were harvested, and the pellet was resuspended in 1 mL of M9 medium and transferred to a precooled 1.5 mL tube. The tube was then centrifuged for 45 s in a cooled microcentrifuge rotor at 12,000 × *g*, and the supernatant was thoroughly aspirated. The pellet was resuspended in 200 μL of M9 medium with 0.2% glucose. The suspension was diluted with the same medium to obtain an OD_600_ of 32, and chloramphenicol was added at a final concentration of 50 μg/mL to stop protein synthesis. Both the cell suspension and labeled substrate dissolved in M9 medium with 0.2% glucose were separately preincubated for 20 min at 37°C. Uptake was initiated by adding the cell suspension to the substrate solution to obtain an OD_600_ of 10. The reaction mixture was incubated at 37°C. Subsequently, 12.5 μL sample fractions were periodically collected and immediately filtered through 0.45-μm GVS North America 13-mm nitrocellulose membranes presoaked in M9 medium on a vacuum manifold, followed by two washes with 1 mL of the same medium. Membranes were air-dried at 37°C for 18–20 h, and radioactivity was measured using 5 mL of GC-106 scintillation liquid (4 g 2,5-diphenyloxazole and 0.1 g 2,2′-(1,4-phenylene)bis(5-phenyl-1,3-oxazole) dissolved in 1 L of toluene) on a RackBeta1215 liquid scintillation spectrometer (LKB, Finland). The amount of radioactivity absorbed by the membrane was used as control. For each substrate concentration, the cell-free reaction mixture was incubated, filtered, washed, and counted as being identical to the experimental reactions. The measured values were then subtracted from those obtained in the appropriate experiment. Transport activity was expressed as nanomoles of substrate taken up by 1 mg dry cellular weight (DCW) in 1 min. The DCW value was calculated based on the OD_600_ of the cell suspension, as described previously (Khozov et al., 2023). All comparisons were performed using two-tailed Student’s *t-*test with unequal variances at a significance level of 5%.

### 2.7 Measurement of *in vivo* luminescence and bacterial growth

A single colony of the assayed strain was inoculated into 5 mL of LB medium supplemented with 100 mg/L ampicillin to maintain the pDEW_*yhjE* plasmid. The overnight culture was diluted to an initial OD_600_ of 0.004 with fresh M9 medium supplemented with 2 g/L glucose and 40 mM l-threonine. Two hundred microliters of the culture were added to each well of a 96-well plate (black-walled, transparent flat bottom; cat. #665096 Greiner Bio-One, Frickenhausen, Germany). The outer wells were not used to avoid edge effects. A well containing a sterile medium was used as a blank. The plates were incubated at 37°C with double-orbital shaking at 600 rpm using a CLARIOstar Plus luminometer (BMG Labtech, Ortenberg, Germany), and OD_600_ and luminescence were measured every 15 min. No luminescence emission filter was used. The photomultiplier gain was automatically controlled using an enhanced dynamic range function. The measured values were normalized to a 1 s accumulation time. The acquired data were analyzed using the MARS software. Blank values were subtracted from the raw OD_600_ and relative luminescence units (RLU) values. The corrected RLU reads at each time point were divided by the corresponding OD_600_ values to normalize the RLU per cell mass for each well. The average RLU/OD_600_ values and standard deviations were calculated and plotted against the OD_600_.

### 2.8 Structural modeling and visualization

The protein structures predicted using AlphaFold 2 (Jumper et al., 2021) were obtained from the AlphaFold Protein Structure Database (Varadi et al., 2022). Structural visualization was performed using UCSF ChimeraX (Pettersen et al., 2021).

### 2.9 Phylogenetic analysis of transport proteins

Evolutionary history was inferred using the maximum likelihood method, and the Whelan and Goldman + Freq. Model (Whelan and Goldman, 2001). The initial tree(s) for the heuristic search were automatically obtained by applying the Neighbor-Join and BioNJ algorithms to a matrix of pairwise distances estimated using the JTT model and then selecting the topology with a superior log likelihood value. A discrete gamma distribution was used to model the evolutionary rate differences among sites [five categories (+*G*, parameter = 8.5853)]. The rate variation model allowed some sites to be evolutionarily invariable ([+*I*], 0.00% sites). Evolutionary analyses were conducted using MEGA11 (Tamura et al., 2021). The phylogenetic affiliations of the transporters were determined according to the Transporter Classification Database (Saier et al., 2014).

## 3 Results

### 3.1 Identification of multicopy suppressors of the growth defect caused by sstT, tdcC, livKHMGF, brnQ, and thrP disruption

To identify permeases exhibiting uptake activity toward threonine on the *∆sstT ∆tdcC ∆brnQ ∆livKHMGF ∆thrP* genetic background, we exploited the inability of the threonine-auxotrophic B1895 strain carrying these mutations to grow on a minimal medium with threonine unless its concentration is increased to 2.5 mM. After electroporation of this strain with a genomic library prepared using the chromosome of the *thrBC sstT tdcC* strain B1426 and the pBR322 vector, we selected transformants that regained the ability to grow on M9 agar supplemented with 1 mM threonine. Sequencing of chromosomal regions within plasmids isolated from the transformants revealed eight candidate genes encoding known or putative membrane proteins. Among these, three encode previously characterized amino acid carriers. The product of *sdaC* is an H^+^/l-serine symporter (Hama et al., 1988; Shao et al., 1994). ProP is an H^+^-dependent transport system specific toward zwitterionic osmolytes such as proline and glycine betaine, which protect cells from osmotic stress (MacMillan et al., 1999). In addition, it translocates a wide range of compounds including taurine, proline betaine, pipecolate, azetidine-2-carboxylate, 3,4-dehydroproline, carnitine, and ectoine (Csonka, 1981; Stalmach et al., 1983; McLaggan and Epstein, 1991; Jebbar et al., 1992; Verheul et al., 1998; MacMillan et al., 1999). AlaE is an H^+^-dependent antiporter that translocates l-alanine from the cytoplasm to the periplasmic space (Kim et al., 2015; Katsube et al., 2019; Ihara et al., 2022). The functions of the remaining five genes, *yqeG, yhjE, ychE, ydgI*, and *yjeM*, are unknown or are controversial, as inferred from literature analysis and the EcoCyc database (Keseler et al., 2017).

### 3.2 Characterization of the multicopy suppressors of threonine uptake deficiency

**Figure 1:**
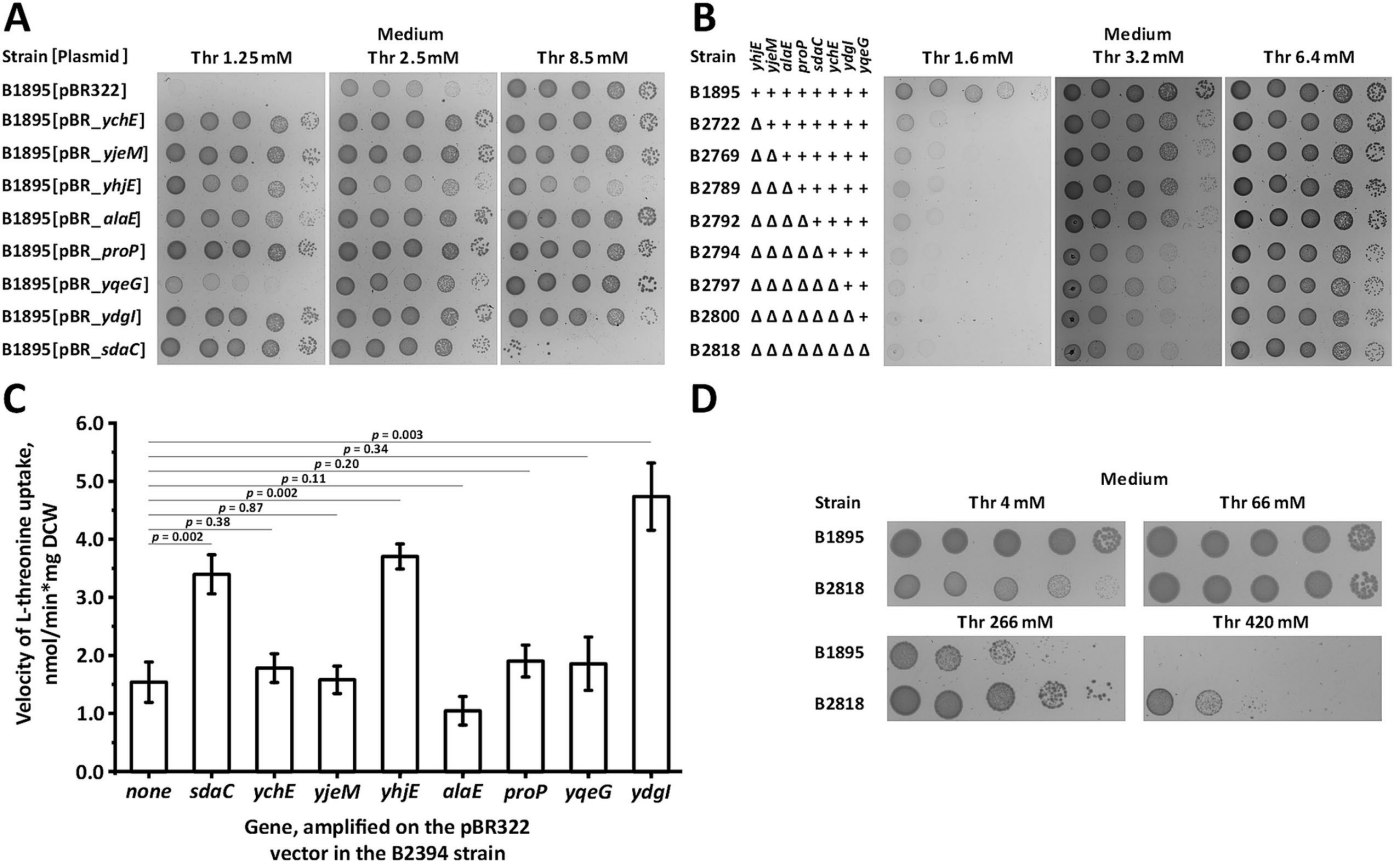
Screening and analysis of the multicopy suppressors of threonine uptake defect. Threonine transport activity was measured as described under “Threonine uptake assay” in the Materials and Methods. “Thr” indicates l-threonine. **(A)** Phenotypic assay of the B1895 threonine-auxotrophic strain and its derivatives carrying pBR-*ychE*, pBR-*yjeM*, pBR-*yhjE*, pBR-*alaE*, pBR-*proP*, pBR-*yqeG*, pBR-*ydgI*, and pBR-*sdaC* plasmids, and the empty pBR322 vector on minimal plates with varying threonine concentrations. **(B)** Comparison of the phenotypes of B1895 and its derivatives lacking incremental combinations of the multicopy suppressors on minimal plates with varying threonine concentrations. **(C)** Comparison of threonine transport activity in the B2394 strain lacking the SstT, TdcC, ThrP, BrnQ, and LIV-I carriers with its derivatives overexpressing SdaC, YchE, YjeM, YhjE, AlaE, ProP, YqeG, and YdgI due to the presence of the corresponding plasmids. Measurements were performed using 800 μM l-threonine, and the reaction time was 1 min. The values shown are the average of three independent biological replicates. Error bars indicate standard deviation. *p-*values were calculated using two-tailed Student’s *t-*test with unequal variances. **(D)** Comparison of the growth fitness of B1895 and its derivative, B2818, lacking eight multicopy suppressors on minimal plates with toxic threonine concentrations.

We verified that the amplification of the eight identified genes, rather than other mutations elsewhere in the genome, suppressed the growth defects of the B1895 strain at non-permissive threonine concentrations. We retransformed B1895 cells with appropriate plasmids and tested the growth of the transformants on minimal medium. All plasmid-carrying strains exhibited significantly improved fitness compared with the parental plasmid-free strain (Figure 1A). Among the multicopy suppressors, *sdaC*, *proP*, *alaE*, *yhjE, ychE, ydgI*, and *yjeM* had similar effects whereas *yqeG* stimulated growth in the medium containing the lowest threonine concentration to a lesser extent. Furthermore, we examined the phenotypes of constructed mutant derivatives of B1895 lacking incremental combinations of suppressors. Inactivation of *yhjE* and *sdaC* caused a prominent growth defect, whereas deletion of other genes resulted in no distinguishable phenotype under the conditions used (Figure 1B). This may be explained by the relatively low contribution of *proP*, *alaE*, *ychE, ydgI, yjeM*, and *yqeG* to the total threonine uptake, which was negligible in the case of wild-type alleles but became significant when these genes were amplified on the multicopy vector. The B2818 strain, which lacked all known threonine permeases and the eight identified suppressors, grew well on minimal plates with 6.4 mM threonine, indicating that it still expresses unidentified transport system(s). The activity of this carrier may also exceed that of the other carriers whose deletions result in no visible growth defects, thereby hiding their phenotype. This may represent a multi-subunit complex whose dedicated genes cannot be retrieved from the genome *en bloc*. Alternatively, the amplification of this system using the pBR322 vector may be deleterious to cell viability. Therefore, other approaches should be used to identify the remaining permease(s).

Unlike the other genes, *sdaC* rendered the B1895 strain sensitive to threonine in the medium at a concentration of 8.5 mM while being overexpressed on the multicopy plasmid (Figure 1A). In this manner, it resembles BrnQ, a high-capacity threonine carrier whose inactivation, based on our data, renders cells threonine-resistant (Khozov et al., 2023). Hence, we presumed that the inactivation of the identified suppressors exerts a similar effect. Indeed, a comparison of the phenotype of the parental B1895 and the B2818 strains lacking all eight suppressors revealed that the latter had significantly improved fitness under toxic threonine concentrations, thereby confirming the reduction of membrane permeability for threonine due to the introduced mutations (Figure 1D).

The observed phenotype of cells carrying the suppressors in the multicopy vector strongly suggests that the products *sdaC*, *proP, alaE, yqeG, yhjE, ychE, ydgI*, and *yjeM* can facilitate threonine translocation under the tested conditions. In addition, the reduced fitness compared with that of the corresponding parent of the strains lacking a chromosomal copy of the suppressor supports the hypothesis that *yhjE* and *sdaC* are involved in threonine uptake. Finally, the sensitivity of the *sdaC-*overexpressing strain to threonine indicates that SdaC can serve as a high-capacity threonine-specific permease. To verify this, we evaluated the threonine uptake in the threonine-prototrophic *∆sstT ∆tdcBCDE::neo ∆thrP ΔbrnQ ∆livKHMGF* mutant B2394 overexpressing the suppressors due to the presence of the corresponding plasmids. In agreement with the phenotypic tests, the strains overexpressing *sdaC, yhjE*, and *ydgI* exhibited an approximately two-fold increase in activity (Figure 1C). Amplification of *ychE, yjeM, alaE, proP*, and *yqeG* did not noticeably affect activity, indicating that their effect on the growth of the threonine uptake-defective strain may occur via an indirect mechanism. However, this phenomenon is most likely due to the requirement of only a slight increase in transport activity to restore growth of the B1895 strain, which is barely detectable via direct activity measurements.

### 3.3 Screening of chromosomal mutations that suppress the growth defect caused the threonine uptake deficiency

**Figure 2:**
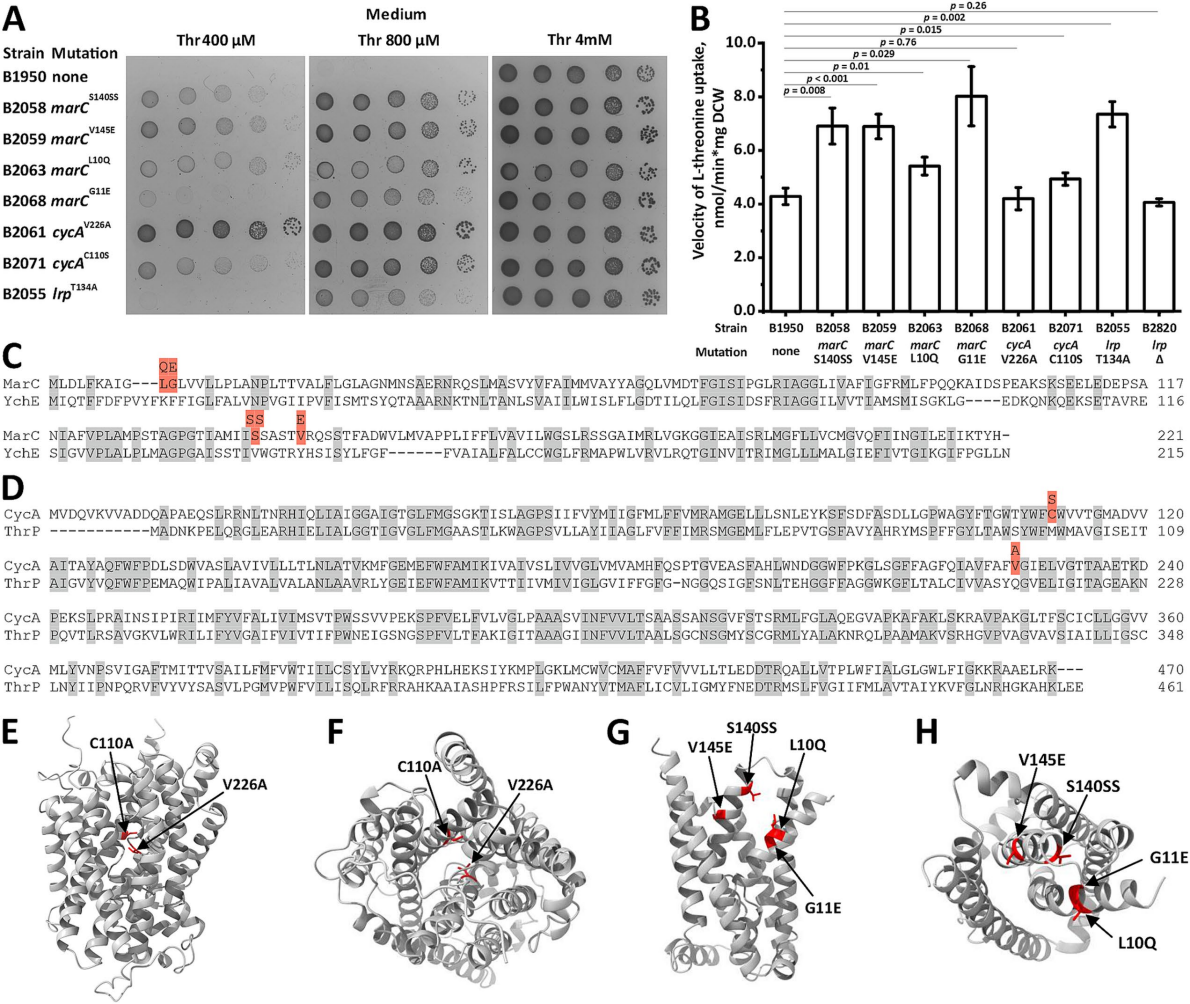
Screening and analysis of chromosomal mutations that suppress threonine uptake defect. Threonine transport activity was measured as described under “Threonine uptake assay” in the Materials and Methods. “Thr” indicated l-threonine. **(A)** Phenotypic assay for the B1950 threonine-auxotrophic strain and its derivatives harboring mutations in *marC, cycA*, and *lrp* loci on minimal plates with varying threonine concentrations. **(B)** Comparison of threonine transport activity in the B1950 strain its derivatives harboring mutations in *marC, cycA*, and *lrp* loci. Measurements were performed using 2.5 mM l-threonine, and the reaction time was 1 min. The values shown are the average of three independent biological replicates. Error bars indicate standard deviation. *p-*values were calculated using two-tailed Student’s *t-*test with unequal variances. **(C,D)** MarC-YchE and CycA-ThrP alignments performed using the Clustal Omega algorithm (Sievers and Higgins, 2014) and UniProt web service (Bateman et al., 2025). The gray and red fillings indicate identical amino acid residues and the amino acid substitutions detected in the mutant derivatives of B1950 that can grow at a non-permissive threonine concentration, respectively. **(E–H)** Ternary structures of CycA and MarC predicted by AlphaFold2 (Jumper et al., 2021). The red filling indicates mutated residues. **(E)** CycA, side view. **(F)** CycA, top view. **(G)** MarC, side view. **(H)** MarC, top view.

Next, we determined whether chromosomal suppressor mutations capable of restoring the growth defects caused by the inactivation of transport systems can be selected. We plated the B1950 strain lacking *sstT, tdcC, thrP, brnQ, livKHMGF, yjeM, sdaC, ychE*, and *ydgI* on M9 plates with 0.4 mM threonine. Under these conditions, B1950 showed no detectable growth, thereby allowing the selection of mutants that regained the ability to utilize exogenous threonine. Genome resequencing of seven independent mutants revealed three distinct sites of the causative mutations (Table 3). The first site is the gene encoding MarC, a protein with unknown function but is associated with isobutanol- and isoprenol-tolerant phenotypes (Minty et al., 2011; Babel and Krömer, 2020). Interestingly, MarC shared some homology with YchE (Figure 2C). We identified four amino acid substitutions, S140SS, V145E, L10Q, and G11E, which improved the growth of the B1950 strain. The second site was *cycA*, in which V226A and C110S substitutions were detected. CycA is a well-known H^+^-dependent symporter that translocates glycine and l-alanine as well as d-alanine, d-serine, *β*-alanine, and an antibiotic d-cycloserine (Robbins and Oxender, 1973; Schneider et al., 2004; Baisa et al., 2013). Recent data indicate that l-valine and *α*-aminobutyrate are also substrates of CycA (Hook et al., 2022). CycA shares homology with the known threonine/serine carrier ThrP (Figure 2D). The last site was *lrp*, which encodes a global transcription regulator that controls more than 10% of *E. coli* genes, including those involved in amino acid uptake (Tani et al., 2002).

**Table 3:** Genomic mutations suppressing the threonine uptake defect in the B1950 strain.

Strain	Mutations (coordinates are in accordance with the U00096.3 genbank entry)	Annotation
B2055	A932994G	*lrp* ^T134A^
	Δ[1299499–130,069]	Deletion of a region spanning *insH21* that encodes the IS5 transposase
B2059	A1618475T	*marC* ^V145E^
	T1475608C	*ydbD* ^I155I^
	C4509536T	R20W substitution in the cryptic *insO* gene
B2071	T4430191A	*cycA* ^C110S^
	IS5 insertion (1,195 bp in length) between C4494217 and T4494218	
	Δ[1299499–130,069]	Deletion of a region spanning *insH21* that encodes the IS5 transposase
B2058	G1618486GGAG	*marC* ^S140SS^
B2061	T4430540С	*cycA* ^V226A^
B2063	A1618880T	*marC* ^L10Q^
B2068	C1618877T	*marC* ^G11E^

Expectedly, all mutants grew faster at restrictive threonine concentrations (0.4 and 0.8 mM) compared with the parental B1950 strain (Figure 2A). The mutations *marC*^V145E^, *marC*^S140SS^, *marC*^L10Q^, and *cycA*^C110S^ provided similar fitness advantages, whereas *marC*^G11E^ and *lrp*^T134A^ stimulated growth to a lesser extent. The B2061 strain harboring *cycA*^V226A^ grew significantly faster than the other mutant strains. When the threonine content in the medium was increased to 4 mM, all mutants and the parental B1950 strain exhibited similar fitness. Direct measurement of threonine uptake revealed that all mutant strains, except B2061, which carried a *cycA*^V226A^ substitution, had significantly increased threonine uptake activity at 2.5 mM (Figure 2B). This inconsistency may be due to the effect of the V226A substitution on the *K*_M_ of CycA toward threonine rather than the *V*_max_ of translocation. In this case, at a substrate concentration close to saturation, no alteration in activity should be observed.

Analysis of the MarC and CycA crystal structures predicted by AlphaFold 2 (Jumper et al., 2021) revealed that the mutations occupied a compact region within both proteins and were located on the surface of the transmembrane helices that constitute the inner interface of the molecules (Figures 2E–H). Hence, the selected amino acid substitutions may affect the substrate binding and specificity of MarC and CycA, thereby allowing them to translocate threonine at a velocity sufficient to support cell growth at threonine concentrations that are non-permissive for the parental strain.

**Figure 3:**
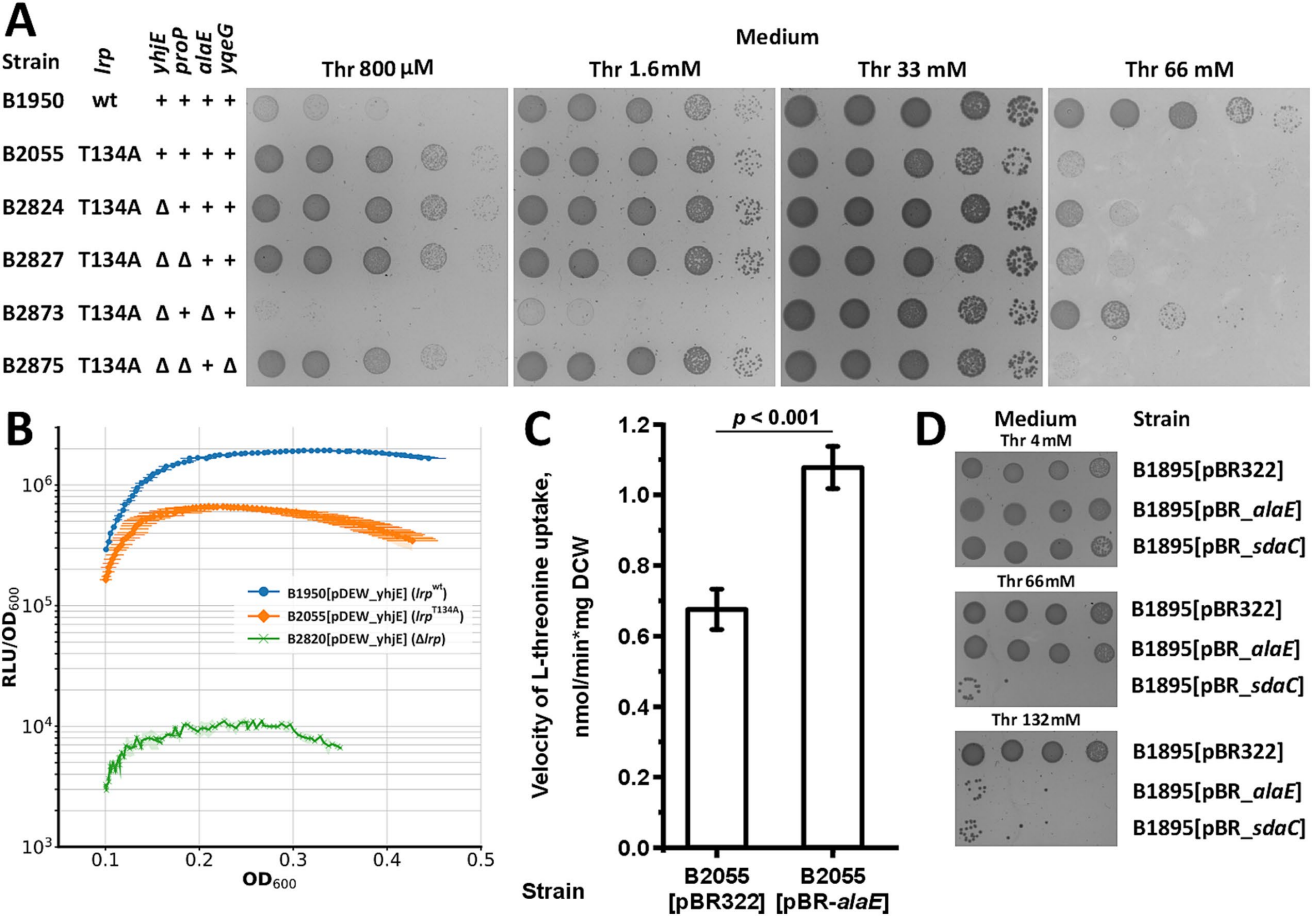
Analysis of the *lrp*^T134A^ mutation phenotype. The threonine uptake measurement and *in vivo* luminescence assays were performed as described under “Threonine uptake assay” and “Measurement of *in vivo* luminescence and bacterial growth,” respectively, in the Materials and Method. “Thr” indicated l-threonine. **(A)** Phenotypic assay for the B2055 threonine-auxotrophic strain carrying the *lrp*^T134A^ allele and its derivatives harboring *yhjE, proP, alaE*, and *yqeG* deletions on minimal plates with varying threonine concentrations. **(B)** Quantification of *yhjE* promoter activity via bioluminescence measurement in strains carrying P*_yhjE_*-*luxCDABE* transcriptional fusion and either the *lrp*^wt^, *lrp*^T134A^, or ∆*lrp* allele. The values shown are the average of three independent biological replicates. Horizontal error bars and filled area indicate standard deviation for OD_600_ and RLU/OD_600_, respectively. **(C)** Evaluation of AlaE activity in the B2055 strain carrying the *lrp*^T134A^ allele. Measurements were performed using 800 μM l-threonine, and the reaction time was 1 min. The values shown are the average of three independent biological replicates. Error bars indicate standard deviation. *p-*values were calculated using two-tailed Student’s *t-*test with unequal variances. **(D)** Comparison of the growth fitness of B1895 and its derivatives overexpressing either AlaE or SdaC on minimal plates supplemented with toxic threonine concentrations.

Next, we investigated the mechanism by which the T134A substitution in Lrp confers a fitness advantage to threonine uptake-defective strains. First, we compared the transport activity of the B2055 strain (B1950 *lrp*^T134A^) with that of its isogenic derivative, B2820, which carries the ∆*lrp* allele. The strain lacking Lrp did not exhibit increased transport activity, whereas B2055 did (Figure 2B). Thus, T134A is not a loss-of-function mutation. Next, we directly assessed the function of Lrp^T134A^. In our preliminary study, we found that the activity of the *yhjE* promoter was strictly dependent on Lrp. This allowed us to estimate Lrp activity using a transcriptional fusion of the *yhjE* promoter and *luxCDABE* operon of *Photorhabdus luminescens* in the stains carrying the *lrp^wt^*, *lrp*^T134A^, or *∆lrp* alleles (Figure 3B). The results indicate that Lrp^T134A^ activates *yhjE* transcription to a slightly lower extent than wild-type Lrp. At the same time, *lrp*^T134A^ confers a phenotype distinct from that of the ∆*lrp* mutation, whose introduction led to a two-fold reduction in *yhjE* promoter activity compared with that in the B1950 strain carrying the wild-type *lrp* allele. Hence, this experiment indicates that consistent with the activity measurement results, *lrp*^T134A^ confers the synthesis of active but altered Lrp. Interestingly, the same mutation has been reported to cause a defect in the activation of the *papBA* operon encoding pyelonephritis-associated pili and shows normal activation of the *ilvIH* operon (Kaltenbach et al., 1998).

Based on this reasoning and the fact that Lrp controls the expression of various proteins involved in amino acid metabolism and transport processes, we presumed that the mutation led to increased synthesis of a transport system specific to threonine. To identify the permease, we inactivated *yhjE*, *proP*, *alaE*, and *yqeG* in B2055 cells (Figure 3A). The disruption of *yhjE* and *proP* had no additional defect on the growth of B2055. Meanwhile, YhjE exhibited a prominent phenotype in the genetic background of the B1895 strain carrying the wild-type *lrp* allele. Consistent with our assumption, the results indicate that unlike B1895, B2055 possesses a primary threonine permease other than YhjE while YhjE makes a minor contribution to total threonine uptake. Indeed, the subsequent disruption of *alaE* led to a drastic reduction in the fitness at limiting threonine concentrations (0.8–1.6 mM). Moreover, unlike B2055 which exhibited sensitivity to threonine at 66 mM, B2873 lacking *alaE* exhibited only slightly reduced fitness under such conditions compared with the parental B1950 strain. We also found that similar to *sdaC, alaE* amplified on the multicopy vector conferred the B1895 strain carrying the wild-type *lrp* allele with sensitivity to threonine (Figure 3D), further confirming that *alaE* exhibits transport activity specific toward threonine and is expressed, to some extent, even in the background of the *lrp*^wt^ allele. Finally, we directly measured transport activity using cells of the B2055 strain carrying the pBR_*alaE* plasmid and found that it had significantly increased activity compared with those of the same strain with the empty vector (Figure 3C), whereas a similar experiment with the B2394 strain carrying *lrp*^wt^ allele did not reveal AlaE activity (Figure 1C). Taken together, these results prove that *lrp*^T134A^ leads to impaired or altered Lrp function, which in turn causes increased *alaE* synthesis and suppression of threonine uptake defects in the B1950 strain.

## 4 Discussion

**Figure 4:**
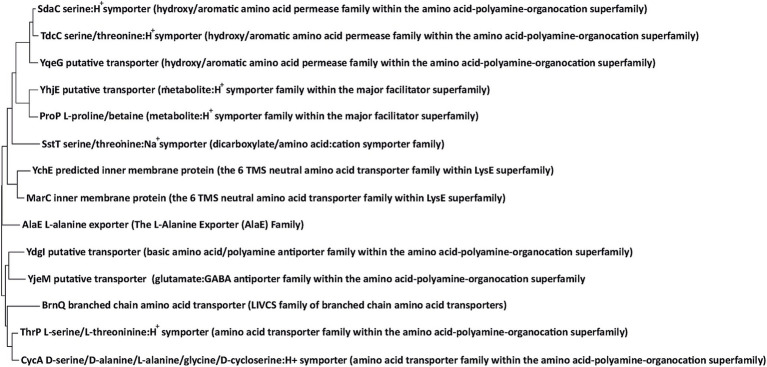
Phylogenic tree of the transport systems involved in threonine uptake. The tree with the highest log likelihood (−13131.09) is shown. The tree is drawn to scale, with branch lengths measured in the number of substitutions per site. All positions with less than 50% site coverage were eliminated. The indicated phylogenetic affiliations of transporters were determined according to the Transporter Classification Database (Saier et al., 2014).

The main purpose of this study was to identify the permeases that mediate threonine uptake in strains lacking the main threonine-specific transport systems SstT, TdcC, ThrP, LIV-I, and BrnQ. Screening for multicopy and chromosomal suppressors identified 10 membrane protein-encoding genes, namely *yhjE, yjeM, ydgI, ychE, marC, yqeG, sdaC, alaE, cycA*, and *proP*, whose amplification on the multicopy vector or specific mutation restored the growth of the threonine uptake-defective strain at non-permissive threonine concentrations. We provided additional evidence for threonine-specific uptake activity for six of the 10 genes. Specifically, the inactivation of *yhjE* and *sdaC* confers further impairment to the fitness of the corresponding mutants compared with the parental strains at restrictive threonine concentrations. This indicates that YhjE and SdaC serve as major threonine-specific carriers in the *sstT tdcC thrP livKHMGF brnQ* strain. Next, the strains overexpressing *yhjE, sdaC, ydgI*, and *alaE* (the last in combination with the *lrp*^T134A^ allele) or carrying the mutant *marC* and *cycA* alleles exhibited significantly increased threonine uptake activity, as revealed by *in vitro* experiments. Finally, *sdaC* and *alaE* overexpression rendered cells threonine-sensitive, suggesting a substantial increase in total threonine intake from the medium. We did not provide such arguments for *yjeM, ychE, yqeG* and *proP*. Thus, these genes may affect the phenotype of the threonine uptake-deficient strain via an indirect mechanism. However, taking into account the observed phenotype of these genes, membrane localization of corresponding proteins, and the phylogenetic relationship between YjeM and YdgI, YchE and MarC, YqeG and SdaC/TdcC, and ProP and YhjE (Figure 4), as well as the intrinsic substrate promiscuity of ProP, we tended to state the threonine-specific transport activity of these proteins, which, nevertheless, are undetectable under our measurement conditions.

All the identified membrane proteins had a minor contribution, if any, to the threonine-specific transport activity in wild-type cells that express the dedicated transport systems SstT, TdcC, ThrP and LIV-I. Therefore, threonine uptake is unlikely to be a function of the carriers relevant to normal *E. coli* physiology. What knowledge can be gained from the results? Notably, the functions of the *yhjE, yjeM, ydgI, ychE, yqeG*, and *marC* genes are unclear. Thus, the obtained data can provide insight into the roles of the listed genes and their products. At this point, we can approach the understanding of YhjE closely. Recently, the *ΔyhjE* mutant has been reported to be defective in the formation of the *bo3* terminal oxidase (Khalfaoui-Hassani et al., 2023). Based on this observation, the authors considered the involvement of YhjE in Cu^2+^ and Fe^2+^ ion transport. Experimental testing of this hypothesis showed that neither of these processes was impaired by *yhjE* inactivation. Therefore, the mechanisms underlying this phenotype remain elusive. In a distinct study, mutation of *yhjE* was reported to render cells resistant to D-valine (Maeda et al., 2020), suggesting that YhjE serves as the main entry point for this compound. It is worth noting, BrnQ and LIV-I, two carriers specific for branched-chain amino acids, can translocate threonine (Khozov et al., 2023). Together with our results, these indicate that the most probable substrates of YhjE are l-isoleucine, l-valine, or l-leucine. Moreover, previous studies have shown that *E. coli* possesses one or more transport systems unrelated to BrnQ and LIV-I (Iaccarino et al., 1978). YhjE may represent one of them; however, further studies are required to confirm this hypothesis.

In our experiments, we identified two genes, *ychE* and *marC*, whose products belong to the 6 TMS Neutral Amino Acid Transporter (NAAT) family (according to the Transporter Classification Database, #2.A.95). Four specific *marC* mutations and *ychE* amplification were found to be capable of suppressing threonine uptake deficiency. Previously, MarC was assumed to be involved in multiple drug resistance (Hächler et al., 1991; Cohen et al., 1993; White et al., 1997) based on its proximity to the *marRAB* operon, which controls the expression of several genes involved in resistance to antibiotics (Keeney et al., 2008; Warner and Levy, 2010), organic solvents (White et al., 1997), oxidative stress, and heavy metals (Alekshun and Levy, 1999). However, a later study reported that inactivation of neither MarC nor YchE affected susceptibility to various antibiotics (McDermott et al., 2008). Different studies have found that non-synonymous single nucleotide polymorphisms and loss-of-function mutations in *marC* increased the tolerance of *E. coli* cells to isobutanol (Minty et al., 2011) and isoprenol (Babel and Krömer, 2020). However, considering the regulatory role of the adjacent *marRAB* operon, whether these phenotypes are related to the function of MarC itself or whether the selected mutations in *marC* alter *marRAB* expression remains unclear. SnatA, another member of the NAAT Family from the hyperthermophilic archaeon *Thermococcus* sp. KS-1 which, when overexpressed in *E. coli*, catalyzes glycine translocation (Akahane et al., 2003), and its activity is inhibited by l-threonine, l-serine, l-alanine, and l-cysteine. Although we cannot exclude the possibility that MarC translocates isobutanol and isoprenol, this fact, together with our results, may suggest that the physiologically relevant substrate range of MarC and YchE includes some of these amino acids or structurally similar compounds. Strains whose growth on minimal medium depends on the function of YchE and mutant MarC can further be used for competitive inhibition assays to identify the exact substrates of these transport systems. The same approach could be applied to *yjeM, ydgI*, and *yqeG*. A literature search and EcoCyc database examination revealed no clues regarding the function of the corresponding proteins, except that YdgI is similar to the arginine:ornithine antiporter ArcD of *Pseudomonas aeruginosa* (Bourdineaud et al., 1993; Herzberg et al., 2006). The ability of these genes, when amplified on a plasmid, to mitigate threonine uptake deficiency and the directly measurable *in vitro* activity of YdgI toward threonine can be used to decipher their physiologically relevant substrates and roles in cellular biology.

The present work extends the substrate range of the SdaC and ProP transport systems. ProP is known to exhibit intrinsic substrate promiscuity and translocates to a wide range of compounds whereas SdaC has been reported to be strictly specific to l-serine and inactive toward threonine (Shao et al., 1994). In contrast to the conclusions of this study, we found that SdaC supports the growth of strains defective in threonine transport at concentrations of 1–3 mM (Figure 4). Moreover, the SdaC-overexpressing strain exhibited threonine sensitivity similar to that of strains expressing the active BrnQ carrier. Thus, SdaC may be a low-affinity and high-capacity threonine-specific permease.

One of the most interesting results was the threonine uptake activity of AlaE. Indeed, we found that *alaE* is a multicopy suppressor of the threonine growth defects in the B1895 strain. Increased *alaE* expression was the exact mechanism by which the B2055 strain carrying the *lrp*^T134A^ allele overcame deficiency in threonine uptake. Next, the wild-type allele on the *lrp*^T134A^ background and amplification of *alaE* on the *lrp*^wt^ background conferred higher sensitivity to threonine in the corresponding strains compared with that of their counterparts lacking the chromosomal *alaE* copy and pBR*-alaE* plasmid, respectively. Finally, the threonine-specific activity of AlaE was directly measured in the strain carrying the pBR_*alaE* plasmid and *lrp*^T134A^. Taken together, these results indicate that AlaE can translocate threonine from the external environment to the cytosol. At the same time, there is strong evidence that AlaE is an H^+^/l-alanine antiporter that operates in the reverse direction and protects cells from toxic l-alanine accumulation (Hori et al., 2011a, 2011b; Kim et al., 2015). These functions are unlikely to be two independent activities in a single transport system. We presume that AlaE is specific to both substrates and can translocate them in both directions, depending on the ratio of their concentrations in the cytosol and external environment, and the magnitude of the electrochemical potential of H^+^ ions on the cytoplasmic membrane.

Finally, the obtained results can be used in metabolic engineering to construct effective threonine-producing strains. We have shown that the strain lacking *yhjE, yjeM, ydgI, ychE, yqeG, sdaC, alaE*, and *proP* as well as *sstT, tdcC, thrP*, and *livKHMGF* exhibit significantly increased fitness under toxic threonine concentrations up to 420 mM compared with its counterpart harboring wild-type alleles of the eight multicopy suppressors. Notably, this phenotype resembles that of strains that overexpress specific threonine exporters, such as RhtA or RhtC (Zakataeva et al., 1999; Livshits et al., 2003), which are commonly used to confer threonine resistance and enhance the ability of producing strains to accumulate threonine (Debabov, 2003; Lee et al., 2007; Dong et al., 2011). Therefore, improving fitness by reducing membrane permeability to threonine could serve as a complementary strategy for constructing industrial strains alongside the overexpression of exporters. Furthermore, it is well established that the ability to accumulate threonine inversely correlates with the threonine-specific uptake activity of cells (Kruse et al., 2001, 2002). The underlying mechanism of this phenomenon may involve the prevention of intracellular threonine accumulation and the subsequent inhibition of key biosynthetic enzymes. Additionally, a producer cell that simultaneously expresses both RhtA/RhtC and threonine-specific uptake systems inevitably translocates threonine in both directions via proton motive force-dependent pathways. This creates a futile cycle that dissipates energy and reduces product yield, which can be mitigated by inactivating the uptake transport systems.

## Data Availability

The original contributions presented in the study are included in the article/supplementary material, further inquiries can be directed to the corresponding author.
